# Monthly Summary

**Published:** 1871-02

**Authors:** 


### MONTHLY SUMMARY.

*Speaking and Singing without a Tongue.*—In the transactions of the Philosophial Society, published between 1742 and 1744, there is an account of Margaret Cutter, who, when four years old, lost her entire tongue from a cancerous affection ; but who, nevertheless, afterward retained the power of taste, swallowing and speech, without any imperfection whatever. She not only spoke as fluently and with as much correctness as other people, but also sang to admiration, articulating with distinctness all her words while singing. What is not less singular, she could form no idea of the use of a tongue in other persons. This remarkable case was brought before the Royal Society, under certificates of attestation from the minister of the parish, a medical practitioner and another respectable citizen, well-known in Suffolk, where she resided. On account of the extraordinary character of the case, the society requested an additional report upon the subject, and from another set of witnesses, named by the society for the purpose, and for whom they drew up the necessary questions and marked out the proper course of examination. The second report coincided with the first in all particulars, and shortly afterward the young woman was brought to London, where she confirmed the accouut by personally appearing, and speaking and singing in the presence of the members of the Royal Society and many other persons.—*The College Courant. Boston Medical & Surgical Journal.*

---

*Pernicious Effects of Mercury Prevented by Sodium.*—The pernicious effects of mercury on the general health of workmen employed in mirror factories is well known. According to recent observations the vapor of this metal is as injurious as its dust. M. Crocks has discovered a means to reduce the deleterious action of the mercury dust; it is only necessary to add to the mercury one-half per-cent. of sodium. This improvement has already been tried in a few factories with the greatest success, and we would recommend manufacturers of mirrors to work their mercury with the addition of the aforesaid proportion of sodium.—*Medical & Surgical Reporter.*

*A Case in Surgery a Hundred Years Ago.*—The following interesting case is reported to us by the venerable Dr, Ebenezer Alden, of Randolph, Mass. The notes were furnished to him by the widow of Dr, Baker :

John Stetson, aged thirty eight, farmer, also accustomed to slaughter cattle, July 19, 1868, in a paroxysm of insanity secreted himself in a lonely place near a swamp, and with a butcher knife made a cæsarean section of his own body, ripping himself open from sternum to pubis. As soon as the deed was done, he came to himself, but could not make his cries for assistance heard. He rolled himself about among the leaves in agony; making also fruitless efforts to reach a neighboring spring, crawling upon the ground and dragging his bowels after him. At length he became exhausted and probably fainted, and thus the bleeding was staunched; so he remained during the night.

In the morning he was found, and life not being extinct, he was placed upon a straw bed and that upon a bier procured from the burying ground, and so conveyed to his house.

Dr. Moses Baker was in attendance, and first bathed the protruding bowels in warm milk and water, carefully cleansing them from the dirt and leaves with which they were covered. He then carefully replaced them within the cavity of the abdomen securing them with sutures, compresses and a bandage. Dr. B. watched over his patient with intense assiduity, making very frequent visits. By his request Dr. Joseph Warren, a distinguished surgeon, who afterwards fell in the service of his country at the memorable battle of Bunker Hill, saw the case, but made no change in the dressings, kindly saying that he could not have dressed the wound more skilfully himself.

On examining the account books of Dr. Baker, now in my possession, I find the last charge for attendance and dressing was on the twenty-fourth day of August, thirty-six days after the injury. On the twenty-sixth there was a charge "for a salve," when it is to be presumed the wound was so nearly healed as to need no further surgical attendance. The recovery was perfect, and the patient was able to labor in the same manner as he had done before the injury. He lived afterwards forty three years until 1811, when he died at the age of eighty-one.—*Medical and Surgical Reporter.*

*Hydrate of Chloral.*—"The hydrate of chloral, about which so much has been written during the past year, has now reached the stage of a quack medicine, and, in the hands of designing or ignorant people, is likely to occasion much mischief. It is sold in fluid form as an anodyne, mixed with gum or sugar water glycerine, or some tincture, and as the strength of the preparation is not given and it is liable to undergo spontaneous decomposition, the patient can never tell how much of a dose he is taking. A bottle of chloral, put up in the usual style of a popular medicine, which was sent to us six months ago for examination has entirely decomposed, and it would be dangerous to use it, as the nature of the products of decomposition are well understood. We must utter a note of warning that it is never safe to take the hydrate of chloral unless freshly prepared and upon prescription of a physician. It is a valuable hypnotic medicine, but is not to be trifled with."—*Scientific American.*

---

*The Effect of the Use of the Sewing Machine* on the health has been investigated by M. Decaisne. of Paris, by observations on 661 work-women. He concludes that "the effects on the locomoter system do not differ from those of excessive exercise of certain limbs to the exclusion of others;" that this form of labor creates no special tendency to indigestions, or uterine affections; and that the machine involves no more danger to health than the needle,

The London Medical Press and Circular supports these conclusions by testimony received from manufacturing districts in England—stating that the sewing machine women suffer less than those laboring in cotton mills and weaving establishments. —*Michigan Univ. Medical Journal.*

---

*Poisoning from the External use of Carbolic Acid.*—A patient in the Montreal General Hospital had suffered for some five weeks with an acute attack of eczema, affecting the entire cutaneous surface. As all other remedies had proved ineffectual, an ointment of carbolic acid and lard, one part to four, was applied to the arms and thighs. An hour and a half after application the patient was found apparently moribund. Prompt cleansing of the surface, and the internal administration of stimulants, by the mouth and rectum, restored him. In five hours afterward, free vomiting took place, the vomited matter having a strong odor of carbolic acid. Upon recovery, the patient stated that a few minutes after the application of the ointment, he realized "a peculiar burning, pricking sensation over the whole body," accompanied by extreme vesical tenesmus, and then followed a state of perfect insensibility.—*Canada Medical Journal.*

---

*Oil of Peppermint as a Local Anæsthetic.*—Dr. A. Wright, writes to the editor of the *Lancet* (Nov. 19, 1870,) that "a few years ago I became acquainted with the fact of the natives [Chinese,] when suffering with facial neuralgia, using oil of peppermint, which they lightly applied to the seat of pain with a camel-hair pencil. Since then, in my own practice, I in the same way frequently employ oil of peppermint as a local anæsthetic, not only in neuralgia, but also in gout, with remarkably good results; indeed, the relief from pain I have found to be almost instantaneous.,"

It is worthy of note that some Chinese pharmaceutists in San Francisco and New York have been selling a remedy for neuralgia, which has gained some repute. It is a liquid put up in very small vials, holding about half a drachm each, which are sold at an exorbitant price. The liquid has a strong smell of peppermint, and is in all probability the oil of that plant.

---

*Hydrate of Chloral and Morphia in Tetanus.*—Dr. Gronemann has published the case of a man of forty-four, who suffered from tetanus in consequence of a shot in the chest. Chloral alone did not relieve the spasms, but when morphia was given with it, the improvement was manifest. The prescription was as follows: Hydrate of chloral, 150 grains; hydrochlorate of morphia, 3 grains; distilled water and simple syrup, of each two ounces: the fourth part night and morning. The man recovered.

---

*Poisonous Indelible Ink.*—There is in the market an indelible ink which, when it comes in contact with the skin produces eruptions and inflammations, and is very dangerous to handle. The chief constituent of the ink is a black, half fluid resin, made from the oriental anacardia nut. The ink is brown, and has an ethereal odor; in this respect it differs from ordinary indelible inks, and may be distinguished from them.—*Med. & Surg. Rep.*

*Rapidity of Mental Transmission in a Nerve.*—Professor Heimholtz has made some new measurements on the rapidity at which excitation is propagated along the motor nerves of man from the brain to the muscles. The ascertained rapidity of the excitation varies between 260 and 292 feet per second, and the rapidity is also found to be greater in summer than in winter. This result led us to a more exact observation of the influence of temperature, which is ascertained by the artificial cooling or warming of the arm. By this means the accelerating influence of a higher temperature has been clearly determined, so that the interval of time between an impulse of the voluntary lower and the corresponding movement of the muscles is greater in winter than in summer.—*Medical and Surgical Reporter.*

